# Engineering fusogenic molecules to achieve targeted transduction of enveloped lentiviral vectors

**DOI:** 10.1186/1754-1611-3-8

**Published:** 2009-06-02

**Authors:** Yuning Lei, Kye-Il Joo, Pin Wang

**Affiliations:** 1 Mork Family Department of Chemical Engineering and Materials Science, University of Southern California, Los Angeles, California 90089, USA

## Abstract

**Background:**

Lentiviral vectors with broad tropism are one of the most promising gene delivery systems capable of efficiently delivering genes of interest into both dividing and non-dividing cells while maintaining long-term transgene expression. However, there are needs for developing lentiviral vectors with the capability to deliver genes to specific cell types, thus reducing the "off-target" effect of gene therapy. In the present study, we investigated the possibility of engineering the fusion-active domain of a fusogenic molecule (FM) with the aim to improve targeted transduction of lentiviral vectors co-displaying an anti-CD20 antibody (αCD20) and a FM.

**Results:**

Specific mutations were introduced into the fusion domain of a binding-deficient Sindbis virus glycoprotein to generate several mutant FMs. Lentiviral vectors incorporated with αCD20 and one of the engineered FMs were successfully produced and demonstrated to be able to preferentially deliver genes to CD-20-expressing cells. Lentiviral vectors bearing engineered FMs exhibited 8 to 17-fold enhanced transduction towards target cells as compared to the parental FM. Different levels of enhancement were observed for the different engineered FMs. A pH-dependent study of vector transduction showed that the broader pH range of the engineered FM is a possible mechanism for the resulted increase in transduction efficiency.

**Conclusion:**

The fusion domain of Sindbis virus glycoprotein is amenable for engineering and the engineered proteins provide elevated capacity to mediate lentiviral vectors for targeted transduction. Our data suggests that application of such an engineering strategy can optimize the two-molecular targeting method of lentiviral vectors for gene delivery to predetermined cells.

## Background

Viral gene delivery using retroviral vectors remains one of the most promising techniques for gene therapy [1,2]. For certain situations, one may prefer to deliver genes in a cell-type specific manner, alleviating the "off-target" effect [3,4]. Thus, many investigations have focused on how to engineer retroviral vectors into targeted gene delivery vehicles [4]. Significant works have been reported in which the viral envelope glycoprotein is engineered to redirect the host tropism by either inserting a targeting ligand or a single-chain antibody [3,5-11]. Another popular strategy for achieving targeted transduction is directing the viral vectors to the target cell by an adaptor molecule [3,12-18]. Although these approaches can generate vectors that recognize specific cells, the modification and binding interference introduced to the envelope protein unavoidably affects the performance of the glycoprotein to mediate transduction [3,19].

Lentiviral vectors, a subfamily of retroviral vectors, have been widely studied for the purpose of gene delivery because of their ability to transduce both dividing and non-dividing cells [20]. Like other retroviral vectors, their integration ability has enabled the vector-transduced cells to maintain a long-term stable expression of transgenes [1,2]. Recently, we developed a novel method to engineer lentiviral vectors that transduce specific cell types by breaking up the binding and fusion functions of the envelope protein into two distinct proteins [21]. Instead of pseudotyping lentiviral vectors with a modified viral envelope protein, our lentiviral vectors co-display a targeting antibody and a fusogenic molecule (FM) on the same viral vector surface. Based on the molecular recognition, the targeting antibody will direct lentiviral vectors to the specific cell type. The binding between the antibody and the cognate cellular antigen will induce endocytosis resulting in the transport of lentiviral vectors into the endosomal compartment. Once inside the endosome, the FM will undergo a conformation change in response to the drop in pH, thereby releasing the viral core into the cytosol [22].

We previously demonstrated that a binding defective version of the alphavirus Sindbis glycoprotein was able to envelope lentiviral vectors to mediate fusion of viral membrane and endosomal membrane, a critical step for transduction [21,22]. Kielian and co-workers had studied the cholesterol dependency of the Sindbis virus and reported several versions of the Sindbis virus glycoprotein that were less dependent on cholesterol for transduction [23]. We report herein that engineering the fusion domain of the binding defective Sindbis glycoprotein can enhance fusion function of this protein to pair with an anti-CD20 antibody (αCD20), hence mediating targeted transduction of lentiviral vectors to CD20-expressing cells. The cellular antigen used in this study is the CD20 protein, whose expression is B cell specific [24]. It has been shown that 90% of non-Hodgkin's lymphomas are CD20-positive [25-27]. CD20 is not usually expressed on either precursor B lymphoid cells or the majority of plasma B cells [27]. Thus, this stage-specific expression pattern makes CD20 an ideal target for therapies against B cell malignancy.

## Results

### Generation of pH-dependent FMs

**Figure 1:**
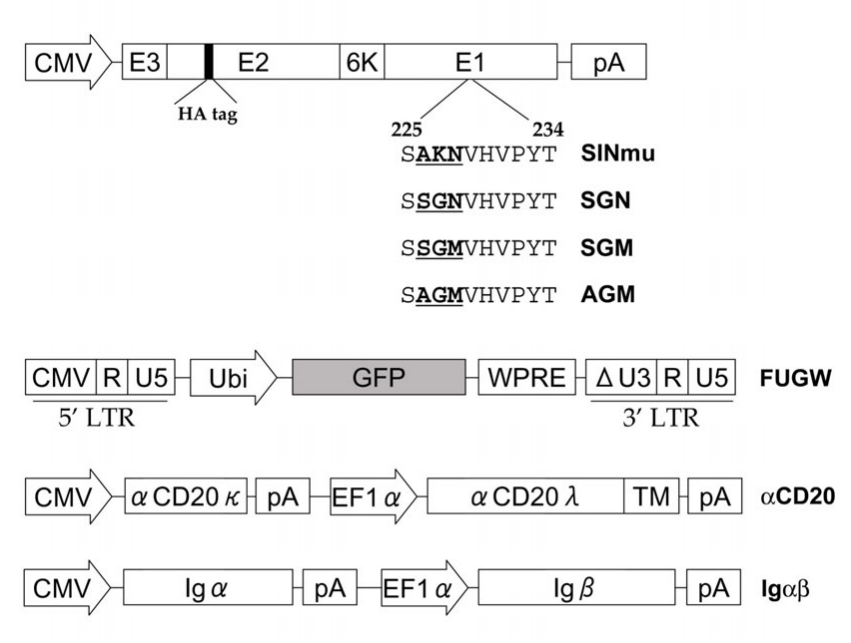
**Schematic representation of key constructs in this study encoding the FM derived from the Sindbis virus glycoprotein, lentiviral transfer vector FUGW, membrane-bound human/mouse chimeric antibody against CD20 (αCD20), and accessory proteins for surface expression of antibody (Igαβ)**. CMV: human cytomegalovirus immediate-early gene promoter; E3: leader peptide of Sindbis virus glycoprotein; E1: E1 protein of the Sindbis virus glycoprotein for mediating fusion; E2: E2 protein of the Sindbis virus glycoprotein for binding to viral receptor; HA tag: 10-amino acid epitope hemagglutinin sequences (MYPYDVPDYA); Ubi: human ubiquitin-C promoter; GFP: green fluorescent protein; WPRE: woodchuck regulatory element; LTR: long-terminal repeat; ΔU3: U3 region with deletion to disable the transcriptional activity of integrated viral LTR promoter; EF1α: human elongation factor 1α promoter; αCD20κ and αCD20λ: light chain and heavy chain of human/mouse chimeric antibody against CD20; TM: human antibody transmembrane domain; Igα and Igβ: human antibody accessory proteins Igα and Igβ. For the FM constructs (SINmu, SGN, SGM and AGM), the amino acid sequences at E1 226 region are shown. The sequence starts at amino acid 225 and ends with amino acid 234 of the wild-type E1 protein. Specific amino acids involved in generating new FMs are shown underlined in bold.

We previously demonstrated that cell-specific targeted transduction can be achieved by lentiviral vectors enveloped with αCD20 and a FM [21]. The FM used in that study was a mutant viral glycoprotein derived from the Sindbis virus. The Sindbis envelope glycoprotein consists of two domains; E1 is responsible for mediating the fusion between the virus and target cell and E2 is responsible for directing the binding of the virus to the cellular antigen on the target cell surface [28]. Chen and co-workers reported a fusion-competent, but binding-deficient form of the Sindbis envelope glycoprotein which was generated by inserting a ZZ binding domain into the E2 region of the envelope protein [13,14]. We further modified this binding-deficient envelope protein by replacing the ZZ binding domain with a HA tag; the resulting protein was designated SINmu [21]. In addition, Kielian and co-workers have previously shown that a mutation on E1, at region 266, of the Sindbis virus envelope protein results in viruses that were less dependent on cholesterol for transduction [23]. Their work identified three distinct mutants with enhanced efficiency to transduce cholesterol-depleted cells [23]. We reasoned that incorporation of these mutations into SINmu might endow new FMs with enhanced efficiency to induce fusion of antibody-displaying lentiviral vectors to accomplish targeted transduction. To test this hypothesis, we generated three FMs designated as SGN, SGM and AGM (Fig. 1). These FMs differed from the parental FM (SINmu) by three amino acids at region 226 of the E1 protein.

### Production of recombinant lentiviral vectors

**Figure 2:**
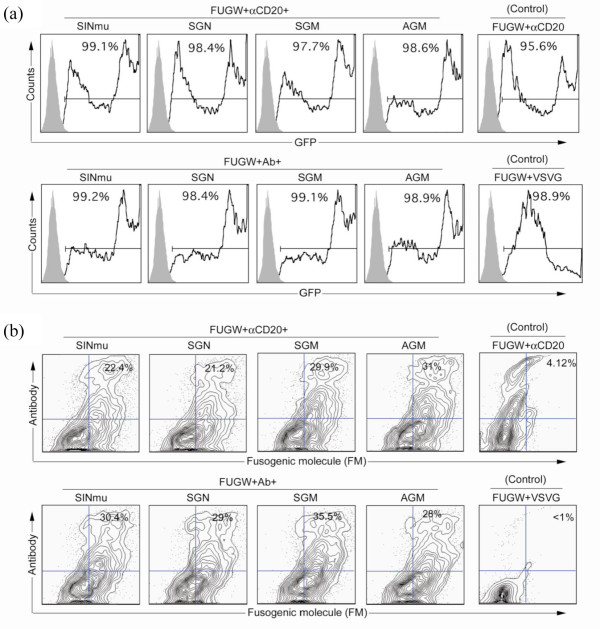
**Co-expression of an antibody and a FM on the surface of transfected vector producing cells**. 293T cells were transiently transfected with plasmids FUGW, pαCD20, pIgαβ, pFM, along with other standard packing plasmids to produce targeted FUGW/αCD20+FM vectors. The plasmid pAb was used in transfection to generate control vectors FUGW/Ab+FM. The transfection without FM plasmid was performed to generate the control vector FUGW/αCD20. The transfection with the standard envelope plasmid encoding VSVG was conducted to generate the non-targeting control vector FUGW/VSVG. (a) FACS analysis of GFP expression in vector producing cells. Solid line, analysis on transfected 293T cells; shaded area, analysis on 293T cells (as control) (b) Gating on GFP-positive cells, co-expression of an antibody and a FM is shown. Expression of an antibody and a FM were detected by using anti-human IgG antibody and anti-HA antibody, respectively.

To evaluate the targeting activity of these engineered FMs, we employed a transient transfection protocol to produce lentiviral vectors enveloped with αCD20 and a specific FM [29]. We co-transfected 293T cells with FUGW, the self-inactivating lentiviral transfer construct that is derived from HIV-1, which contains an internal human ubiquitin-C promoter driving the expression of GFP reporter gene (Fig. 1) [30]. In addition to FUGW, we supplied the lentiviral packaging plasmids, the αCD20 construct, the antibody accessory protein construct, and the plasmid encoding the FM (SINmu, SGN, SGM, or AGM). The αCD20 system has been previously established in our laboratory and has been shown to display on the surface of lentiviral vectors for targeted transduction of CD20-expressing cells [21]. Therefore, we used this as a model system for testing FMs. Antibody accessory proteins (Igα and Igβ) were required for functional expression of the antibody onto the surface of producing cells for its subsequent incorporation into the viral vector (Fig. 1). As a non-targeting control, a transfection was done to prepare a vector pseudotyped with VSVG since it has fairly broad specificity and can transduce a variety of cell types [31]. Negative controls included vectors bearing a FM and an antibody (Ab) that is blind to CD20 antigen and the vector bearing αCD20 only. Two days after transfection, expressions of GFP, αCD20 and FM were analyzed by flow cytometry. Virtually all of the transfected, virus-producing 293T cells expressed GFP which was encoded in FUGW (Fig. 2a). Among these GFP-positive cells, approximately 21–31% of them expressed both αCD20 and a FM (FUGW+αCD20+FM, Fig. 2b, top left). The viral vectors produced by these transfections were designated FUGW/αCD20+FM. A similar percentage of GFP-positive cells were observed to express both a FM and Ab (FUGW+Ab+FM, Fig. 2b, bottom left) and the corresponding viral vectors were designated FUGW/Ab+FM. As expected, the FM signals were not detected on cells transfected to produce αCD20-bearing, but FM-lacking, FUGW/αCD20 vector (FUGW+αCD20, Fig. 2b, top right), and no signals of the FM and the αCD20 were seen on cells producing the FUGW/VSVG vector (FUGW+VSVG, Fig. 2b, bottom right). In addition, it appeared from the expression pattern of FM-transfected cells that the expression level of the four FMs were similar, suggesting that they could be incorporated into the surface of lentiviral vectors with similar efficiency.

### Co-incorporation of the αCD20 and the FM on lentiviral vectors

**Figure 3:**
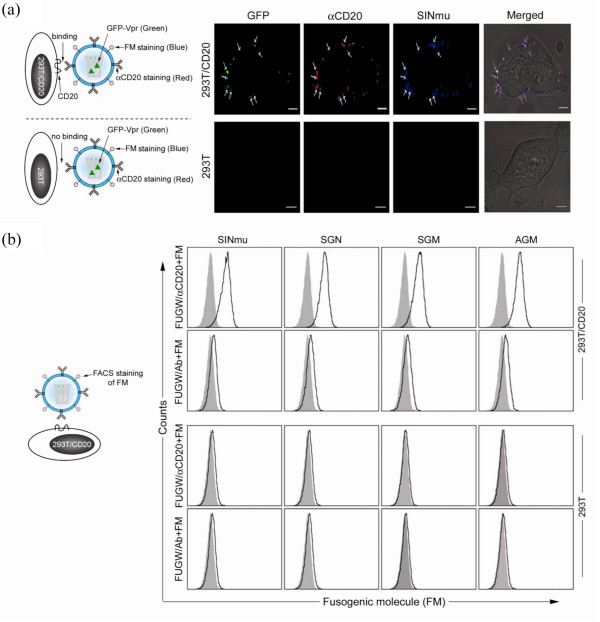
**Co-expression of αCD20 and a FM on the viral vector surface**. (a, left) Schematic diagrams to illustrate the interaction between αCD20 on the vector surface and CD20 on the cell surface, and the immuno-fluorescent staining scheme. (a, right) Acquired confocal images of labeled viral vector binding to cells. (b) FACS analysis of 293T or 293T/CD20 cells incubated with FUGW/αCD20+FM or FUGW/Ab+FM. The binding of virus to 293T/CD20 cells was detected by FACS staining with antibody against the FM. Solid line, analysis on cells incubated with indicated viral vectors; shaded area (control), analysis on cells without incubation with vectors.

In order for our targeting system to work, lentiviral vectors must be enveloped with both αCD20 for binding, and a FM for fusion. We employed a confocal imaging method to analyze the co-incorporation of the αCD20 and the FM. The target cells were incubated with viral vectors at 4°C for 1 hour, followed by sequential staining of the FM (blue color) and the αCD20 (red color). To label the core of the vectors, we adapted a previously reported method to synthesize viral vectors encapsulated with a protein (GFP-Vpr) consisting of GFP fused with viral protein R (Vpr) [32]. It has been shown that the provision of GFP-Vpr in *trans *during transfection can allow the fluorescent protein to be incorporated into the core of HIV-based lentiviral vectors through the interaction between Vpr and the P6 region of the HIV gag protein [32]. We harvested GFP-Vpr-tagged lentiviral vectors bearing αCD20 and SINmu and incubated them with 293T/CD20 cells at 4°C for 1 hour. After extensive washing, the treated cells were subjected to immuno-fluorescence staining and imaging. GFP-labeled signals were detected on the surface of 293T/CD20 cells and their signals were co-localized with signals for both αCD20 and SINmu (Fig. 3a, top), while no fluorescence signals were obtained for 293T cells lacking the expression of CD20 (Fig. 3a, bottom). The co-localization of GFP, αCD20 and SINmu suggested that the cells can produce lentiviral vectors displaying both αCD20 and a FM in a single virion. Similar results were also observed for vectors bearing the other type of FMs (SGN, SGM, or AGM) (data not shown).

We conducted flow cytometry analysis of cells incubated with viral vectors to further examine their surface properties. Various vectors (FUGW/αCD20+FM) were allowed to bind to 293T/CD20 target cells at 4°C for 1 hour, after which the FM was stained and analyzed. Only the vectors bearing both αCD20 and a FM could bind to 293T/CD20 cells and be detected by using an antibody against the FM. Therefore, flow cytometry signals can indicate the presence of both αCD20 and a FM on the same viral surface. As shown in Fig. 3(b), no detectable FM signals were seen when the 293T cells were incubated with either FUGW/αCD20+FM or FUGW/Ab+FM vectors, indicating that the lentiviral vectors were unable to bind to 293T cells lacking the expression of CD20. When FUGW/Ab+FM were incubated with 293T/CD20 cells, no signals were detected for the FM (Fig. 3b). Clear signals were obtained when 293T/CD20 and FUGW/αCD20+FM were incubated together (Fig. 3b). This result confirmed that cell-virus binding was attributed to the interaction between the αCD20 and CD20, and both a FM and αCD20 can be incorporated onto the same viral surface.

### Targeted transduction of lentiviral vectors to CD20-positive cell line

**Figure 4:**
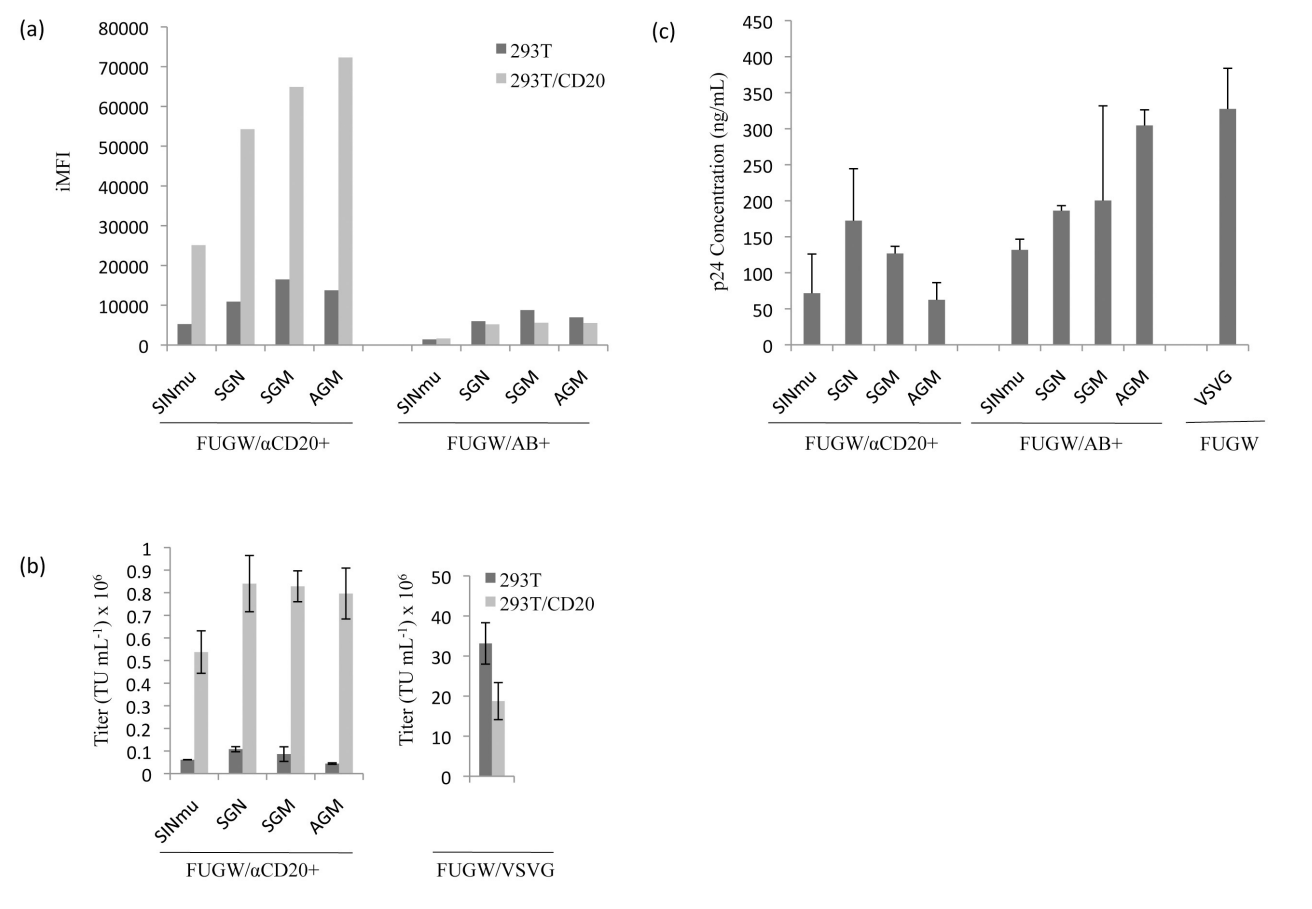
**Transduction of engineered lentiviral vectors bearing both an antibody and FM to cell lines**. 293T and 293T/CD20 cells (2 × 10^5^) were transduced with 2 ml of fresh viral vectors (FUGW/αCD20+FM, or FUGW/Ab+FM). Transduction of 293T cells was included as control. (a) iMFI on 293T and 293T/CD20 cells transduced by the fresh viral vectors (FUGW/αCD20+FM, or FUGW/Ab+FM). (b) Titers of fresh viral vectors (FUGW/αCD20+FM) on 293T and 293T/CD20 cells. (c) p24 amount of fresh viral vectors (FUGW/αCD20+FM, FUGW/Ab+FM, and FUGW/VSVG)

We then investigated how lentiviral vectors bearing different FMs would transduce cells by using 293T/CD20 as the target cell line, and the 293T parental cell line as the negative control. Six days post-transduction, cells were analyzed by flow cytometry. To quantify the difference of our targeted transduction system, we utilized a metric that incorporates both the efficiency and magnitude of the GFP signals detected from the transduced cells [33]. The transduction magnitude was obtained by the mean fluorescence intensity (MFI) of the transduced cells. By multiplying the MFI by transduction efficiency, we derived a metric termed integrated MFI (iMFI) that reflects the total intensity of the GFP signals from the virus transduced cells. As indicated in Fig. 4(a), FUGW/αCD20+AGM displayed the highest iMFI in the target cells (293T/CD20), followed by FUGW/αCD20+SGM, FUGW/αCD20+SGN, and FUGW/αCD20+SINmu. When the same set of viral vectors were used to transduce the non-target cells (293T), much lower iMFI signals were detected. The specific transduction titers of these viral vectors against 293T and 293T/CD20 cells were measured in Fig. 4(b); 8-17-fold increase of preferential transduction of CD20-expressing cells was achieved, depending on which FM was used (Fig. 4b). To confirm that the specific transduction was mediated by the incorporated antibody on the vector surface, we made control vectors bearing a FM and Ab. Spin-transduction of these vectors to 293T/CD20 and 293T cells showed low iMFI signals (Fig. 4a). In addition, to eliminate the possibility that the difference was due to a variance in viral production from the producing cells, we performed an enzyme linked immunosorbent assay (ELISA) to detect the p24 levels in the viral supernatants. As indicated in Fig. 4(c), the p24 levels between the different lentiviral vectors were in the similar range. When comparing the ability of various FMs to mediate transduction, the FM with higher efficiency for targeted transduction would always result in higher background transduction.

### pH Dependence of various FMs

**Figure 5:**
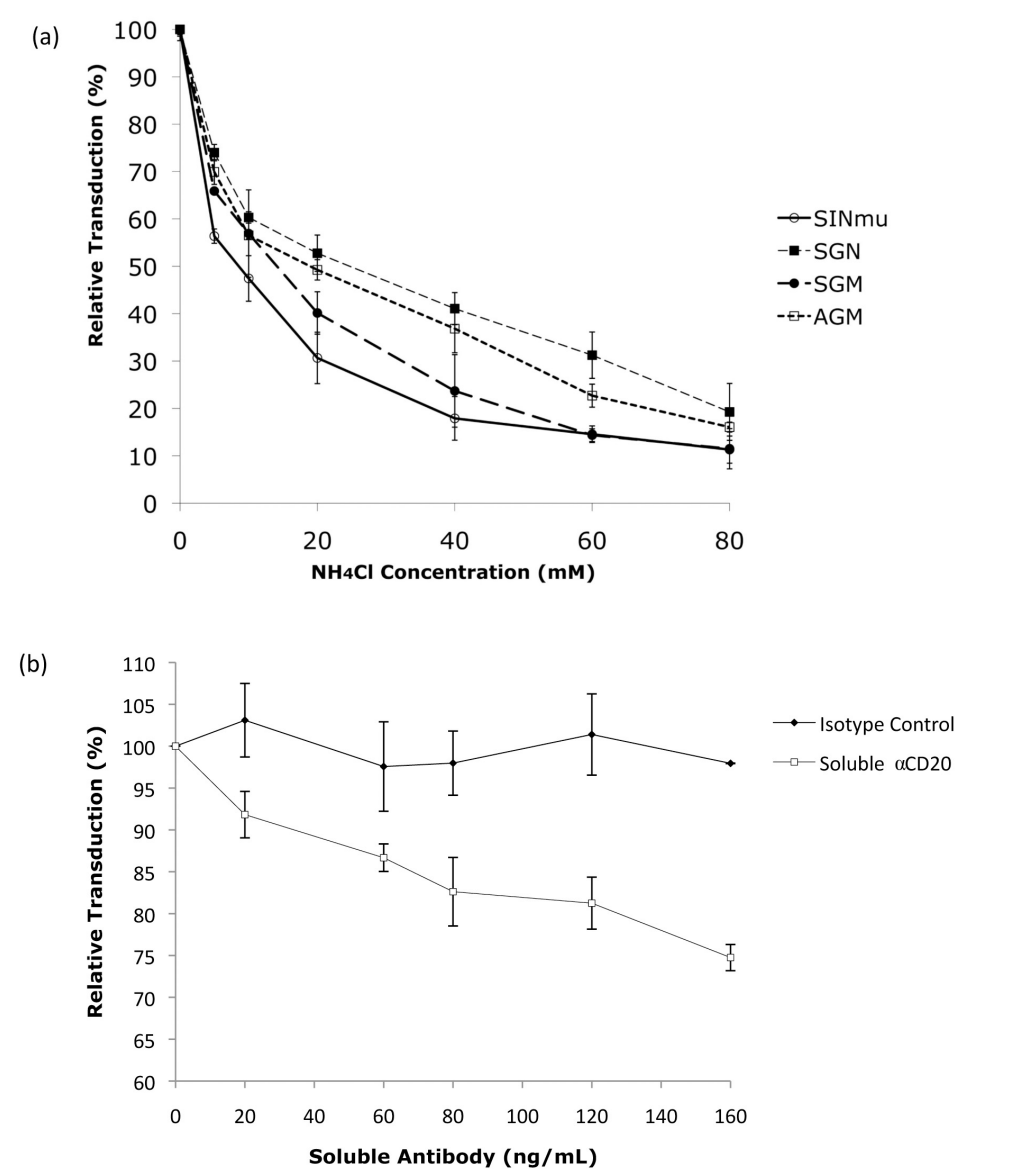
**Examination of addition of NH_4_Cl or solubleαCD20 on the targeted transduction results**. (a) NH_4_Cl was added into indicated viral supernatants during transduction for 8 hours, after which, the supernatants were replaced with fresh media. GFP expression was analyzed 3 days post-transduction. (b) Various amount of soluble αCD20 or isotype control were added into indicated viral supernatants during transduction for 8 hours, after which, the supernants were replaced with fresh media.

Our transduction experiment clearly showed the important role of FMs in determining the overall vector infectivity and that the newly engineered FMs exhibited improved ability to induce targeted transduction as compared to original FM (SINmu) (Fig. 4). We designed an experiment to investigate the possible underlying mechanism responsible for their differences. Lentiviral vectors, FUGW/αCD20+SINmu, FUGW/αCD20+SGN, FUGW/αCD20+SGM, and FUGW/αCD20+AGM, were incubated with 293T/CD20 cells in the absence or presence of a graded concentration of ammonium chloride (NH_4_Cl); NH_4_Cl is known to be able to neutralize the acidic endosomal environment [34]. The changes in transduction were measured by flow cytometry. As shown in Fig. 5(a), the four vectors displaying various FMs behaved differently in response to different concentration of NH_4_Cl. FUGW/αCD20+SINmu was the most sensitive to the neutralization treatment and transduction efficiency dropped the fastest as a result of the changes in pH environment, followed by FUGW/αCD20+SGN, FUGW/αCD20+SGM, and FUGW/αCD20+AGM. It appeared that the engineered vector that was more resistant to the NH_4_Cl treatment could transduce target cells with a higher efficiency.

### Antibody directed targeted transduction

To confirm that the targeted transduction was triggered by the specific interaction between the antibody and the cognate antigen, we performed an antibody competition assay. Lentiviral vector, FUGW/αCD20+SGN was incubated with 293T/CD20 cells in the presence of various concentration of either soluble αCD20 antibody or an isotype control (Fig. 5b). We found that the targeted transduction efficiency decreased as the concentration of soluble αCD20 antibody increased. When the isotype control was used, no decrease in transduction efficiency was observed. This suggested that the soluble αCD20 competed with the targeted lentiviral vector leading to a decreased uptake of the vectors.

### Targeted transduction of lentiviral vectors to unfractionated primary B cells

**Figure 6:**
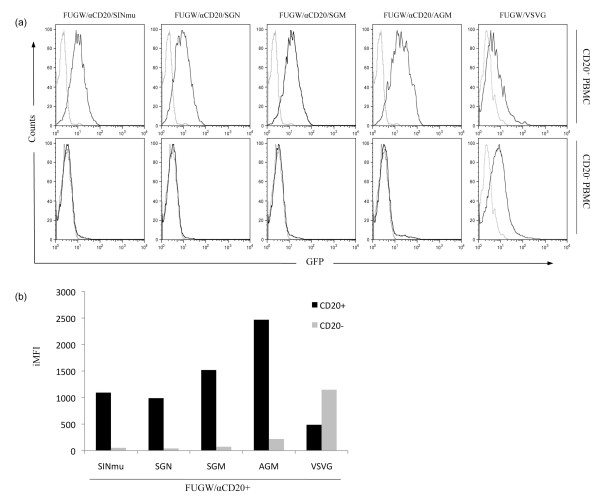
**Targeted transduction of CD20-positive human primary B cells**. (a) Fresh unfractionated human PBMCs (1 × 10^6^) were spin-transduced twice with indicated vectors (FUGW/αCD20+FM, 2.5 × 10^6 ^TU, or FUGW/VSVG, 25 × 10^6 ^TU). 48 hours later, cells were collected and analyzed by FACS (b) iMFI on fresh unfractionated human PBMCs transduced by the indicated viral vectors (FUGW/αCD20+FM, and FUGW/VSVG).

One of the advantages of using targeted vectors is their potential ability to transduce specific cell types in a mixed population without the need to isolate the target cells. We tested whether our targeted vectors with engineered FMs can specifically transduce primary B cells in an unfractionated primary cell population. One million of fresh, unfractionated human peripheral blood mononuclear cells (PBMC) were transduced twice with concentrated FUGW/αCD20+FM (2.5 × 10^6 ^TU), or FUGW/VSVG (25 × 10^6 ^TU). The cells were analyzed by flow cytometry 2 days post-transduction. As shown in Fig. 6(a), transduction of bulk PBMC populations with targeted vectors resulted in specific modification of CD20-positive PBMC, whereas no GFP signals were detected in CD20-negative cells. In the control experiment where FUGW/VSVG was used for transducing bulk PBMC populations, GFP signals were detected in both CD20-positive and CD20-negative cells and a higher iMFI signal was detected in the CD20-negative PBMC as compared to CD20-positive PBMC. Quantification of total expression intensity indicated that vector FUGW/αCD20+AGM attributed the highest iMFI in the CD20-positive PBMC, followed by FUGW/αCD20+SGM, FUGW/αCD20+SIN, and FUGW/αCD20+SGN (Fig. 6b). This was in good agreement with the specific transduction against 293T/CD20 cells (Fig. 4b). The stable integration of the GFP gene was confirmed by the genomic PCR analysis (data not shown).

## Discussion

Lentiviral vectors pseudotyped with envelope glycoprotein from other genus of viruses, such as VSVG, have shown to be able to genetically modify cells with great efficiency and broad tropism [31]. However, certain applications may require using vector systems with cell-type specificity [3,4]. Previous studies have shown that by breaking up binding and fusion functions into αCD20 and a FM, targeted transduction can be accomplished [21].

To test whether engineered FMs can improve vector targeting efficiency, we transduced 293T/CD20 with freshly collected viral supernatants containing targeted lentiviral vectors. Higher iMFI was observed when lentiviral vector, FUGW/αCD20+AGM, was used, followed by a decrease in iMFI in FUGW/αCD20+SGM, FUGW/αCD20+SGN, and FUGW/αCD20+SINmu, respectively (Fig. 4a). These results suggested that FUGW/αCD20+AGM was capable of transducing target cells with higher efficiency and transgene expression. However, some background levels of transduction on non-target cells were observed. This is likely resulted from the endocytosis induced by non-specific cellular receptors, although we did not observe obvious binding between targeting vectors and non-target cells (Fig. 3b). We then set to examine whether such targeted vectors can achieve similar results in PBMC. As shown in Fig. 6(a), a similar trend was observed in that only CD20-expressing PBMC were shown to be specifically transduced by concentrated virus, whereas no transduction was observed in the CD20-negative population. In the control experiment, targeted lentiviral vectors were substituted with a non-targeted vector, FUGW/VSVG, resulting in detection of GFP in all cell types, regardless of CD20-expression. It is known that FUGW/VSVG preferentially transduce CD20-negative populations as compared to CD20-positive population [35]. Furthermore, a higher MOI was needed to achieve similar transduction efficiency highlighting the advantage of targeting. In agreement with previous cell line data, lentiviral vector, FUGW/αCD20+AGM, was able to attribute the highest iMFI followed by a decrease in iMFI in FUGW/αCD20+SGM, FUGW/αCD20+SINmu, and FUGW/αCD20+SGN respectively (Fig. 6a).

In order for our targeting system to work, the virus producing cells must co-express both an antibody and a FM. These vectors can then bind to the target cells based on the recognition provided by the displayed antibody. When adding a soluble antibody, transduction was inhibited whereas addition of the isotype control had no effect on transduction, indicating the binding requirement for targeted transduction (Fig. 5b). After binding, these vectors are endocytosed and transported to the endosomal compartments. The changing of the pH environment within the endosomes triggers the FM to alter its conformation, leading to fusion between the endosomal membrane and the viral membrane [22]. We believe these steps are required for a successful targeted transduction and employed various methods to investigate this targeted transduction pathway. We first analyzed our virus producing cells for the display of the αCD20 and the FM by flow cytometry (Fig. 2b), followed by confocal microscopic imaging to confirm the co-incorporation of both molecules in a single viral vector (Fig. 3a). GFP-Vpr fusion protein was used to label the viral core, while Alexa594-conjugated and Cy5 antibodies were used to detect the αCD20 and the FMs, respectively. Co-localization of all three colors was observed, indicating that the viral vector carried both the αCD20 and the FM. Cell-virus binding assay was employed to evaluate whether the incorporated antibody can provide the function to direct the viral vectors to the target cells (Fig. 3b). As shown by both flow cytometry analysis and confocal imaging, only the lentiviral vectors bearing the targeting antibody were able to bind to the target cells.

Since our engineered lentiviral vectors were enveloped with αCD20 and a FM, it was conceivable that optimization of these two proteins could potentially improve the efficiency of this targeting system. Kielian and co-workers generated three different mutants of the Sindbis virus glycoprotein and found that these mutants could increase the cholesterol independence of the Sindbis virus [23]. We adapted these mutations into the original FM (SINmu) to generate three new FMs (SGN, SGM, or AGM) (Fig. 1). We found that these new FMs exhibited enhanced ability to mediate lentiviral vectors to transduce the target cells. We further analyzed the responsiveness of these FMs to acidic neutralization. The neutralization assay with vectors bearing different FMs revealed that the original FM (SINmu) was the most sensitive to the pH change in the endosome, as the infectivity of the vector bearing SINmu dropped the fastest with respect to the ammonium chloride treatment. The new FMs responded slower than that of SINmu to the treatment, with AGM being the least pH-sensitive FM (Fig. 5a). These results were consistent with the transduction assay, in which the vector bearing AGM had the highest transduction titer (Fig. 4b).

The fusion mechanism for the Sindbis glycoprotein involves a drop in pH that triggers a conformational change in the E2 and E1 subunits that exposes the previously hidden E1 hydrophobic domain. The E1 domain then interacts with cholesterol in the target membrane which leads to the fusion of the viral and cellular membranes [36]. Similar to the hemagglutinin glycoprotein of the Influenza virus, mutations throughout the trimer interface alter the pH threshold required for fusion by either destabilizing the buried position of the fusion peptide or by modifying salt bridges and hydrogen bonding between trimer subunits [37]. We speculate that these mutations may destabilize the fusion loop of the E1 domain which lowers the required activation energy needed for fusion. Since the lentiviral vectors are required to escape from the endosome in order to transduce the host cells, the FM that is active throughout a wider pH range could endow the corresponding lentiviral vector with a larger window to escape from the degradation pathway.

## Conclusion

In summary, we have demonstrated in this report that targeted transduction could be accomplished by enveloping lentiviral vectors with αCD20 and a FM. One of the major advantages of this system is the flexibility as the vectors can be readily reengineered to target different cell type. As shown in our previously published data, our system was capable of targeting antigen specific immunoglobulins by simply swapping the display antibody with an antigen [38]. We are currently expanding our targeting strategy to a variety of cell types including gp160 expressing cells. We have also demonstrated that this targeted system is amenable for further optimization to improve efficiency. Although higher transduction efficiency could be achieved by pairing the recognition antibody with novel engineered FMs to improve fusion properties, it also led to higher background transduction efficiency. We are currently studying our engineered FM in the hope that we would gain some insight in the fusion process allowing us to design a better FM capable of enhanced targeted transduction with decreased background transduction. When comparing findings from this paper to our previously published data [39], it becomes apparent that a judicious choice must be made when it comes to the FM, since FMs behave differently under different settings. In the case of gamma-retrovirus pseudotyped with FM, one FM showed enhanced transduction whereas in the case of lentiviral vector, multiple FMs showed enhanced efficiency. It is noteworthy that one advantage of using lentiviral vector as compared to gamma-retroviral vector lies in the ability of lentiviral vectors to transduce non-dividing cells.

## Methods

### Construct preparation

The original FM, SINmu, was previously constructed in our laboratory [21]. It was generated by replacing amino acids 157KE158 with 157AA158 of the E2 protein of the Sindbis virus glycoprotein. Additional deletion was performed to remove amino acids 61–64 in the E3 protein of the Sindbis virus glycoprotein. We also inserted a hemagglutinin epitope tag sequence (MYPYDVPDYA) between amino acids 71 and 74 of the E2 proteins for detection purpose (Fig. 1). Based on SINmu, we performed 4-primer PCR-mutagenesis to generate mutants SGN, SGM, and AGM. To construct SGN, a forward primer (BsiW1fw, 5'-GCC AGA TGA GTG AGG CGT ACG TCG AAT TGT CAG C-3') and a backward primer (SGNbw, 5'-CAT GCA CGT ACC GGA GGA AGG CTT GAG TAG CCT AAT GTC TGT GCT G-3') were used to amplify the E1 domain containing the BsiW1 site and the desired SGN mutations at the E1 226 region. In parallel, a forward primer (SGNfw, 5'-AGC CTT CCT CCG GTA ACG TGC ATG TCC CGT ACA CGC AGG CC-3') and a backward primer (Mfe1bw, 5'-GCT GCA ATA AAC AAG TTA ACA ACA ACA ATT GCA TTC ATT TTA TG-3') were used to amplify the E1 domain containing the desired SGN mutations at the E1 226 region and Mfe1 site. The DNA products from these two reactions were PCR-assembled using BsiW1fw and Mfe1bw as the primer pair and cloned into pcDNA3 (Invitrogen) to yield SGN. The similar PCR protocol was used to generate mutant SGM and AGM, except that the primers SGNbw and SGNfw were replaced by (SGMbw, 5'-CAT GCA CCA TAC CGG AGG AAG GCT TGA GTA GCC TAA TGT CTG TGC TG-3') and (SGMfw, 5'-AGC CTT CCT CCG GTA TGG TGC ATG TCC CGT ACA CGC AGG CC-3') for construction of SGM, and by (AGMbw, 5'-CAT GCA CCA TAC CGG CGG AAG GCT TGA GTA GCC TAA TGT CTG TGC TG-3') and (AGMfw, 5'-AGC CTT CCG CCG GTA TGG TGC ATG TCC CGT ACA CGC AGG CC-3') for construction of AGM.

The construct encoding the membrane bound form of the human/mouse chimeric antibody against human CD20 antigen (pαCD20) and the construct encoding the human antibody accessory proteins Igα and Igβ (pIgαβ) were constructed previously in our laboratory [21]. Briefly, the cDNAs of the human κ light chain constant region, and the membrane bound human IgG1 constant chain region were amplified and inserted downstream of the human CMV and EF1α promoters, respectively, in the pBudCD4.1 vector (Invitrogen). The light chain variable region from the murine αCD20 was amplified from an αCD20 hybridoma cell line (ATCC, Manassas, VA, HB-9303) with (CD20Lvfw, 5'-CCC AAG CTT ATG GAA ACC CCA GCG CAG CTT C-3') and (CD20Lvbw, 5'-CAG CCA CCG TAC GTT TCA GCT CCA GCT TG-3') and inserted directly upstream of the light chain constant region via Hind3 and BsiW1 restriction sites. In parallel, the heavy chain variable region was amplified with (CD20Hvfw, 5'-GGA CTC GAG ATG GAG TTT GGG CTG AGC TG-3') and (CD20Hvbw, 5'-GGT GCT AGC TGA AGA GAC GGT GAC CGT G-3') and inserted directly upstream of the heavy chain constant region via Xho1 and Nhe1 restriction sites. The non-relevant antibody (Ab) used in this study, B12, was kindly provided by the laboratory of Dr. Dennis Burton at the Scripps Research Institute [40]. The membrane bound B12 antibody was constructed similarly to the αCD20, except that (B12Lvfw, 5'-CCC AAG CTT ACC ATG GGT GTG CCC ATC C-3') and (B12Lvbw, 5'-CAC CGT ACG TTT CCT CTC CAG TTT GGT CCC-3') were used to amplify the light chain variable region, and (B12Hvfw, 5'-GGG ACC AAA CTC GAG AGG AAA CGT ACG GTG-3') and (B12Hvbw, 5'-GGT GCT AGC TGA GCT CAC GAT GAC CGT GG-3') were used to amplify the heavy chain variable region. The HIV-1-based lentiviral transfer plasmid FUGW was constructed by the laboratory of Dr. David Baltimore at the California Institute of Technology [30].

### Cell line construction

The 293T cell line was obtained from ATCC. The cell line 293T/CD20 was generated by stable transduction of vesicular stomatitis virus glycoprotein (VSVG) enveloped lentiviral vector encoding the cDNA for the human CD20 protein.

### Virus production

Recombinant lentiviral vectors were generated via the standard calcium phosphate precipitation technique [29]. The vectors producing 293T cells were seeded in a 6-cm culture dish with DMEM medium supplemented with 10% Fetal Bovine Serum (Sigma, St. Louis, MO), L-glutamine (10 mL L^-1^), penicillin (100 units mL^-1^), and streptomycin (100 units mL^-1^). After 16–18 hours, when the confluency was about 80%, the seeded 293T cells were transfected with plasmid DNAs. The packaging plasmids, pMDLg/pRRE (2.5 μg) and pRSV-Rev (2.5 μg) [38], the plasmids for surface display of αCD20, pαCD20 (2.5 μg) and pIgαβ (2.5 μg), the plasmid encoding the FMs, pFM (2.5 μg), and the lentiviral transfer plasmid FUGW (5 μg) were mixed with calcium chloride and added drop-wise to 2× HBS solution with constant vortexing [41]. The lentiviral vector FUGW/Ab+FM was produced similarly with the exception that the plasmid encoding pAb was used instead of pαCD20. Co-transfection of 293T cells with the lentiviral transfer plasmid FUGW (5 μg), the packaging plasmids pMDLg/pRRE (2.5 μg) and pRSV-Rev (2.5 μg), and the envelope plasmid pVSVG (2.5 μg) was performed to generate the lentiviral vector FUGW/VSVG. Transfected cells were replenished with pre-warmed fresh media 4 hours post-transfection. 48 hours later, viral supernatants were harvested, and filtered through a 0.45 μm pore size filter (Nalgene, Rochester, NY).

### Fluorescent labeling

GFP-Vpr-labeled lentiviral vectors were produced by co-transfecting 293T cells with the plasmid encoding GFP-Vpr [22] in addition to the plasmids used in generation of corresponding lentiviral vectors. For imaging cell-virus binding, 5 × 10^5 ^cells were seeded onto a 35 mm glass-bottom culture dish (MatTek Corporation) and grown at 37°C overnight. The seeded cells were rinsed with cold PBS twice and incubated with concentrated viral vectors for 1 hour at 4°C to allow for binding. Cells were washed with cold PBS to remove unbound viral vectors, fixed for 10 minutes on ice using 4% formaldehyde, and then immunostained by Alexa594-conjugated anti-human IgG (Molecular Probes) and biotin-conjugated anti-HA antibody (Miltenyi Biotec Inc.), followed by a secondary staining with Cy5-conjugated streptavidin (Zymed Laboratories). The images were acquired with a Zeiss LSM-510 laser scanning confocal microscope (Carl Zeiss, Thornwood, NY) using a plan-apochromat 63×/1.4 oil immersion objective. The images were processed using the LSM 510 software version 3.2 SP2.

### FACS analysis of cell-virus binding

0.5 million cells (293T or 293T/CD20) were incubated with 2 mL of various lentiviral vectors at 4°C for 1 hour. Cell-virus complexes were then washed with 4 mL of cold PBS and spun down at 2,000 rpm for 5 minutes. Cell-virus complexes were then stained with anti-HA antibody to detect the presence of the FM on the lentiviral vectors bound to the target cells. After staining, cells-virus complexes were analyzed by flow cytometry (BD Bioscience) to measure the binding.

### p24 analysis of lentivral vectors

Various lentiviral vectors (10 μL, fresh supernatant) were lysed with 90 μL of 10% Triton-X 100 in PBS and the p24 levels were measured by a p24 antigen capture enzyme linked immunosorbent assay (ELISA) kit (ImmunoDiagnostics, Woburn, MA)

### Targeted transduction

Various lentiviral vectors (2 mL, fresh supernatant) were added to 0.2 million cells (293T or 293T/CD20) plated in a 24-well culture dish and spin-transduced for 90 minutes at 2,500 rpm and 25°C. The medium was then removed and replenished with 2 mL fresh media. Treated cells were incubated for 6 days at 37°C and 5% CO_2_. The percentage of GFP expression was determined by flow cytometry analysis. The titer was calculated based on the GFP expression in the viral dilution range where the percentage of GFP-positive cells linearly corresponded to the volume of virus.

### NH_4_Cl neutralizing assay

293T/CD20 cells (0.2 million) were incubated with lentiviral vectors (FUGW/αCD20+FM) in the presence of various amounts of NH_4_Cl in a 24-well culture dish at 37°C and 5% CO_2 _for 8 hours. The medium was then removed and replenished with fresh medium and incubated for an additional 3 days prior to flow cytometry analysis.

### Antibody competition assay

293T/CD20 cells (0.2 million) were incubate with lentiviral vectors (FUGW/αCD20/SGN) in the presence of various amounts of either soluble αCD20 or isotype control in a 24-well culture dish at 37°C and 5% CO_2 _for 8 hours. The medium was then removed and replenished with fresh medium and incubated for an additional 3 days prior to flow cytometry analysis.

### Targeted transduction of unfractioned PBMC

Lentiviral vectors (FUGW/αCD20+FM) were generated and concentrated by ultracentrifugation (Optima L-90K Ultracentrifuge, Beckman Coulter, Fullerton, CA) at 25,000 rpm for 90 minutes and resuspended in 100 μl cold PBS after overnight incubation. The concentrated vectors (2.5 × 10^6 ^transduction units (TU) of the targeted lentiviral vectors and 25 × 10^6 ^of the VSVG pseudotyped lentiviral vector) and 1 million unfractioned PBMC were spin-transduced for 90 minutes at 2,500 rpm and 25°C. The medium was then removed and replenished with 1 mL fresh medium. LPS (50 μg ml^-1^) was then added to support the survival and growth of B cells. Treated cells were collected 48 hours post-transduction and stained with anti-human CD20 antibody. Flow cytometry was employed to determine the efficiency of targeted transduction.

## Competing interests

The authors declare that they have no competing interests.

## Authors' contributions

YL designed the experiments, performed the experiments, analyzed the data, and wrote the paper. YL and KJ carried out the fluorescent labeling. PW designed the experiments, analyzed the data, and wrote the paper.
